# A Rare Case of Embryonal Rhabdomyosarcoma of the Uterine Cervix

**DOI:** 10.1155/2022/8459566

**Published:** 2022-04-13

**Authors:** Vishal Bahall, Lance De Barry, Steven Sankar

**Affiliations:** ^1^Department of Obstetrics and Gynaecology, San Fernando General Hospital, South-West Regional Health Authority, Trinidad and Tobago; ^2^Department of Radiology, San Fernando General Hospital, South-West Regional Health Authority, Trinidad and Tobago

## Abstract

Embryonal rhabdomyosarcoma (RMS) of the uterine cervix is an exceedingly rare mesenchymal tumor that accounts for less than 1% of all cervical cancers. This highly malignant tumor primarily affects adolescents and young adults. Due to the paucity of publications on this clinical entity, there are no clearly established treatment protocols. However, a multimodal approach to treatment that involves surgical intervention combined with adjuvant chemoradiotherapy appears to improve patient outcomes. Herein, we report a case of embryonal rhabdomyosarcoma of the uterine cervix in a 24-year-old female, who presented with an exophytic cervical mass and vaginal bleeding. Histopathology and immunohistochemistry confirmed embryonal rhabdomyosarcoma of the uterine cervix with extension into the lower uterine segment. This patient was successfully managed with a combination of neoadjuvant chemoradiotherapy, a total abdominal hysterectomy with bilateral salpingo-oophorectomy, and adjuvant chemoradiotherapy.

## 1. Introduction

Embryonal rhabdomyosarcoma (RMS) is the most common soft tissue sarcoma occurring in the prepubertal age group [1]. This mesenchymal tumor arises from immature striated skeletal myocytes and can occur anywhere in the head and neck, lymph nodes, extremities, retroperitoneum, and trunk [2]. The genitourinary tract is the second most common site involved after the head and neck [2]. Embryonal RMS typically affects the vagina or bladder in infants and the uterus or cervix in women of reproductive age [2].

Embryonal RMS of the uterine cervix presents with vaginal bleeding and an exophytic, polypoid cervical mass [3]. Patients often report pelvic pain, pressure, and urinary or bowel symptoms. Sarcoma botryoides is the most common subtype of embryonal RMS [4]. The diagnosis is confirmed on histopathology and immunohistochemistry with the presence of rhabdomyoblasts within a myxomatous stroma and positive immunohistochemical stains for vimentin, desmin, or actin [4].

Embryonal RMS of the cervix responds best to a multimodal approach to treatment, inclusive of surgical intervention, and systemic chemotherapy with considerations for radiotherapy [5]. Fertility preservation is one major challenge affecting the choice of therapy. Embryonal RMS is a chemosensitive tumor and responds best to the vincristine, adriamycin, and cyclophosphamide (VAC) regime [6]. In this report, we describe a case of embryonal RMS of the uterine cervix occurring in a 24-year-old woman. She presented to us with a prolapsed, exophytic cervical mass and vaginal bleeding. A biopsy revealed an embryonal rhabdomyosarcoma–botryoid variant. Pelvic MRI demonstrated the involvement of both the cervix and lower uterine segment. The patient was successfully managed with neoadjuvant chemoradiotherapy, followed by a total abdominal hysterectomy, bilateral salpingo-oophorectomy, and adjuvant chemoradiotherapy.

## 2. Case Presentation

A 24-year-old virgo-intacta woman presented to her gynecologist with reports of abdominal pain, nocyclical vaginal bleeding, urinary frequency, and incontinence for several months. The pain was described as cramping and poorly localized to the suprapubic area. She denied experiencing fever, weight loss, vaginal discharge, gastrointestinal, and urinary symptoms. She had no prior medical or surgical history, and her gynecological history was unremarkable. The patient also had no personal or family history of cancer.

**Figure 1:**
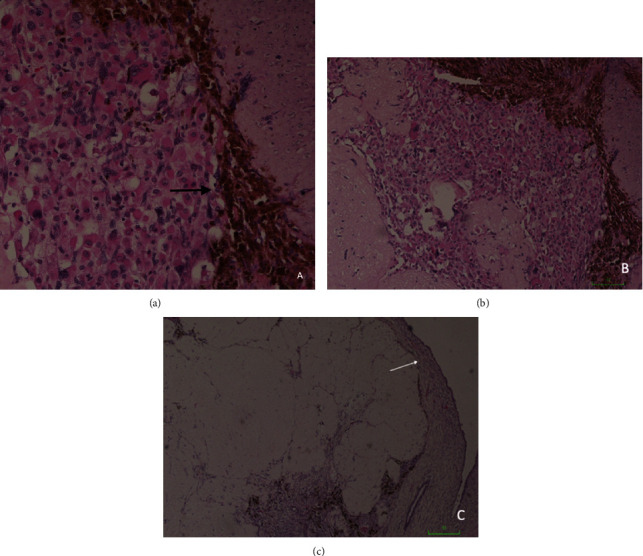
Histopathology and immunohistochemistry of the cervical lesion (a) Tumor cells stain positive for desmin (black arrow). (b) Small, spindled, and round cells interspersed with strap cells against a background of myxoid stroma suggestive of rhabdomyoblastic differentiation. (c) Cambium layer (white arrow) demonstrating foci of subepithelial condensation of undifferentiated cells.

Clinical examination revealed a fungating mass seen extending out of the introitus. The mass measured approximately 6 cm × 5 cm and was located on the anterior lip of the cervix. Blood investigations demonstrated microcytic anemia (hemoglobin 8.8 g/dL, MCV 74.5 fL) and normal renal and liver function tests. Tumor markers such as cancer antigen (CA-125 and CA-19-9), carcinoembryonic antigen (CEA), and alpha-fetoprotein (AFP) were all within normal parameters. The patient subsequently underwent an examination under anesthesia with an excisional biopsy of the cervical mass. Histopathology (Figure 1) revealed primitive mesenchymal elements arranged in both hypo and hypercellular areas with loose myxoid stroma and perivascular condensation. A cambium layer was observed which was composed of subepithelial condensations of undifferentiated cells. The cells were small, spindled-shaped, and contained interspersed strap cells. Immunohistochemistry demonstrated a positive stain for desmin but negative stains for vimentin and actin. These findings were suggestive of embryonal rhabdomyosarcoma of the cervix–botryoid variant.

**Figure 2:**
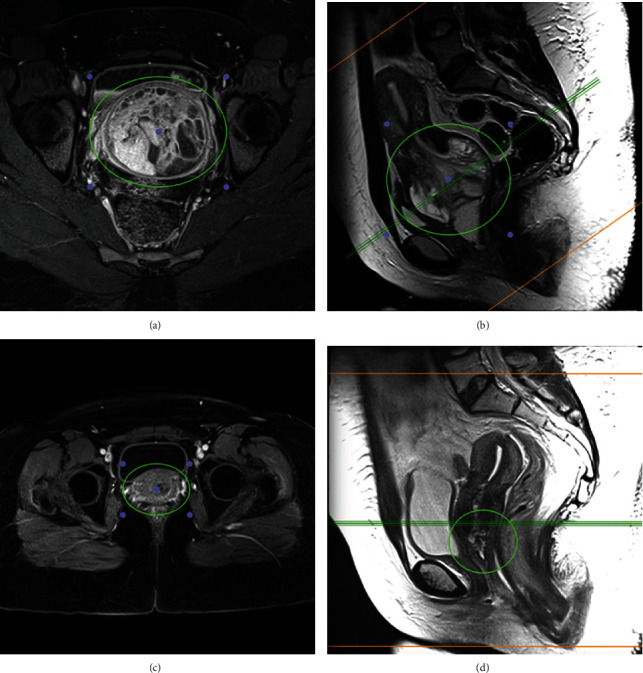
(a, b) MRI of the pelvis (axial and sagittal views) before neoadjuvant chemoradiotherapy demonstrating a heterogeneously enhanced well-circumscribed lesion measuring 3.2 cm(ap) × 6.1 cm(tr) confined within the middle compartment of the pelvis. There is association with distention of the forniceal and transitional compartments of the vagina with caudal displacement of the introitus. (c, d) MRI of the pelvis after neoadjuvant chemoradiotherapy demonstrated a significant decrease in the volume of the lesion which measured 1.3 cm(ap) × 1.2 cm(tr) remaining confined within the middle compartment of the pelvis. The residual disease is epicentred in the upper third of the vagina with cranial extension into the cervical and lower endometrial canal.

Considering these findings, a magnetic resonance imaging (MRI) scan of the abdomen and pelvis was requested to delineate the extent of the disease (Figure 2). Pelvic MRI demonstrated a 6.1 cm × 3.2 cm low T1/high T2 STIR signal in the region of the vaginal vault with associated inferior displacement of the anterior aspect of the cervix and vaginal introitus. A homogenous high T2 signal measuring 0.7 cm was noted in the endometrial canal suggesting endometrial involvement. There was no descent of the urinary bladder to suggest a cystocele. Additionally, the periurethral and paraurethral ligaments as well as the puborectalis muscle appeared normal. The uterus and bilateral ovaries appeared unremarkable. There was also no pelvic, inguinal, or para-aortic lymphadenopathy, adnexal masses, or abdominopelvic free fluid noted.

**Figure 3:**
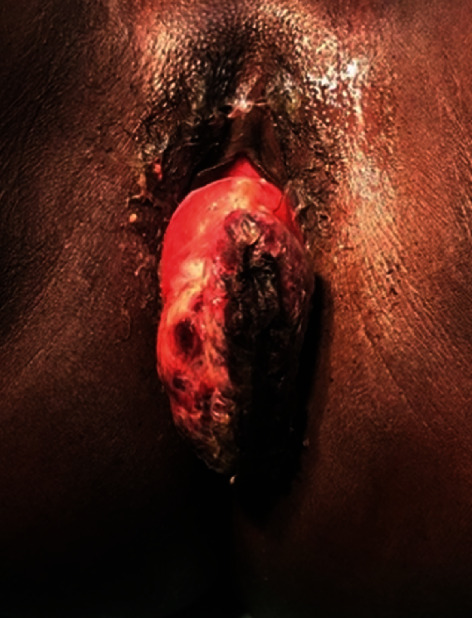
Prolapsed, exophytic cervical mass measuring 8 cm × 8 cm which redeveloped after initial excisional biopsy.

The patient was referred to the Gynecologic Oncology team for further management. Clinical assessment one month after initial excisional biopsy revealed regrowth of the fungating cervical mass with extension beyond the introitus (Figure 3). The patient's case was discussed at the multidisciplinary team (MDT) meeting, and a decision was made for a staging computed tomography (CT) scan of the chest, abdomen, and pelvis followed by neoadjuvant chemoradiotherapy. The staging CT scan demonstrated a 6.1 cm × 3.2 cm mass in the region of the vaginal vault involving the anterior aspect of the cervix with no distant metastasis, and no pelvic, inguinal, or para-aortic lymphadenopathy. Positron emission tomography (PET) CT scan was not available at our institution at that time. The patient received external beam radiotherapy and a chemotherapeutic regime (VAC/IE) consisting of vincristine, adriamycin, and cyclophosphamide on day one, followed by ifosfamide plus etoposide twenty-one days later, for four cycles.

An MRI scan was repeated after the completion of neoadjuvant treatment (Figure 2). Comparison made to pretreatment imaging demonstrated a good response with a significant reduction in the size of the tumor. The residual tumor now measured 4.7 cm × 1.2 cm × 1.3 cm and occupied the cervical canal. Extension into the lower endometrial canal and proximal vagina were noted. There was no parametrial invasion, and the bladder and rectum appeared unremarkable. Additionally, there was no distant metastasis, pelvic, inguinal, or para-aortic lymphadenopathy observed. Considering the reduction in tumor size following neoadjuvant treatment and lack of parametrial and lymph node involvement, a decision was made to proceed with a simple total abdominal hysterectomy and bilateral salpingo-oophorectomy.

Intraoperatively, a 4 cm mass was noted extending from the cervix while the intra-abdominal viscera, uterus, and ovaries appeared grossly unremarkable. The uterus, bilateral fallopian tubes, and ovaries, together with the cervix containing the cervical tumor, was successfully removed without any intraoperative complications. Histopathology demonstrated a 4.7 cm tumor that extended from the upper cervix to involve the lower uterine segment and ectocervix. The tumor was composed of mainly hypocellular areas with rhabdoid changes and myxoid stroma. Immunohistochemistry again demonstrated a positive stain for desmin and negative stains for vimentin and actin. These findings confirmed embryonal rhabdomyosarcoma of the uterine cervix with local invasion into the lower uterine segment. A partial response to neoadjuvant treatment was noted. The margins were negative, and lymphovascular invasion was not identified.

The patient's postoperative course was unremarkable. She was subsequently enrolled for adjuvant chemotherapy consisting of the previous VAC/IE regime and vaginal brachytherapy. Follow-up CT scans of the chest, abdomen, and pelvis demonstrated no signs of recurrence or metastasis. The patient is currently 12 months since surgery, and she is well and disease-free. She is receiving follow-up care in both the Gynecologic Oncology and Medical Oncology clinics.

## 3. Discussion

Embryonal rhabdomyosarcoma (RMS) accounts for 0.4-1.0% of all cervical cancer cases [2]. Epidemiological data suggest that up to 90% of cases occur in women less than 25 years old, and approximately 70% occur in children less than 12 years of age [7]. Perimenopausal women are infrequently affected, and the prognosis in this age category is generally poor [7].

The most common histologic subtype of RMS affecting the female genital tract is embryonal which accounts for 60% of all cases [1]. Less common subtypes include alveolar and pleomorphic. Furthermore, there are three subtypes of embryonal RMS–sarcoma botryoides, spindle cell, and anaplastic [8]. The most common variant of embryonal RMS is sarcoma botyroides which is also associated with a more favorable prognosis compared to the other histological subtypes [8].

The pathogenesis of embryonal RMS of the cervix remains unclear; however, several reports implicate germline mutations involving the DICER1 gene. The DICER1 gene codes for endoribonuclease which has an important role in the biogenesis of microRNAs and the control of protein translation [9]. An analysis by Apellaniz-Ruiz et al. suggests that almost all cases of gynecologic embryonal RMS may harbor DICER1 alterations [9]. This pathologic germline variation in DICER1 may create a predisposition to the hereditary cancer syndrome–DICER1 syndrome, characterized by the development of multiple benign and malignant tumors [9, 10]. Additionally, inactivating mutations of the p53 tumor suppressor gene located on chromosome 17 have also been described [10]. Genetic evaluation with counselling and/or testing for the DICER1 gene mutation may help identify an underlying tumor predisposition [9]. Our patient did not seek genetic counselling due to a lack of available genetic services in our clinical setting.

Patients with embryonal RMS of the cervix typically present with vaginal bleeding and an exophytic or polypoid cervical mass [3]. The tumor often resembles a grape-like cluster with an average size of 5.75 cm (range: 2.0 cm to 9.5 cm) [5]. The tumor may also prolapse past the introitus, such as in the case described. For the unsuspecting gynecologist, this may be overlooked as a cervical polyp or leiomyoma. Depending on the size of the tumor, patients may also report urinary incontinence, constipation, and symptoms of pelvic pain or pressure [3].

The diagnosis of embryonal RMS is established purely on histopathology with the aid of immunohistochemistry. Histologic features include the presence of rhabdomyoblasts on a background of myxoid stroma [4]. Specifically, sarcoma botyroides is identified by a distinct “cambium layer” found beneath the epithelium, which is composed of a dense zone of undifferentiated cells [5]. Histologic differential diagnoses for sarcoma botyroides include adenosarcoma, rhabdomyoma, and cervical mesodermal polyps [4]. Immunohistochemistry has an important role in tumor identification since it is difficult to diagnose embryonal RMS without confirmation of myogenic differentiation. The sarcoma botryoides subtype of embryonal RMS demonstrates a stain positive for desmin, vimentin, and actin, which are components of striated skeletal muscle [10]. In our case, histologic samples demonstrated a positive stain for desmin thus indicating a myogenic origin.

MRI is currently the gold standard imaging modality used to delineate the disease extent, assess for local invasion, identify distant metastases, and plan surgical intervention [3]. A staging system has been proposed by the Intergroup Rhabdomyosarcoma Study Group (ISRG) according to tumor size, primary site, local invasion of surrounding tissues, lymph node involvement, and distant metastasis [11].

Currently, there is no consensus regarding an optimum management protocol for embryonal RMS. However, a multimodal approach to treatment appears to improve patient outcomes. This consists of a combination of surgical intervention, systemic chemotherapy, and considerations for radiotherapy [5]. Fertility preservation is one major challenge influencing treatment choice due to the young age of patients affected. Localized cervical disease is best treated with fertility-sparing procedures such as polypectomy and simple or radical trachelectomy, combined with adjuvant chemotherapy [10, 12]. A simple total hysterectomy with ovarian conservation is recommended for patients with combined cervical and uterine involvement [6, 12]. In the absence of parametrial involvement, radical hysterectomy is not warranted as it is associated with increased morbidity and no improvement in patient outcomes [13]. Our patient had both cervical and uterine involvement with no evidence of parametrial invasion which necessitated a simple total hysterectomy. Lymphadenectomy is not routine and is reserved only for patients with high-risk clinical features [14].

Embryonal sarcoma botryoid RMS of the uterine cervix is a chemosensitive tumor [6]. Currently, combination therapy with vincristine, adriamycin, and cyclophosphamide (VAC) is the gold standard chemotherapeutic regime [6, 15]. In cases of recurrent or refractory disease, the addition of ifosfamide and etoposide (IE) has shown an overall response rate of 69% [15]. However, there are no clear guidelines regarding the optimum dose, and the number of cycles required. The use of neoadjuvant chemotherapy has demonstrated success in reducing the size of large tumors before surgical intervention [6, 10]. The use of neoadjuvant treatment in our patient produced a good clinical and radiological response after the initial MRI showed combined cervical and uterine involvement as well as rapid regrowth of the cervical tumor following excisional biopsy. Adjuvant chemotherapy effectively treats lymphatic micrometastases and is recommended even for IRSG category 1, regardless of the type of surgical intervention performed [6]. Radiotherapy may be warranted for residual tumors based on margin status and lymph node involvement; however, there are no standardized treatment protocols available [6, 11].

According to Elsebaie et al., the overall 5-year survival rate is 65% [14]. Although some patients may be cured with combined surgical and adjuvant treatment, the risk of recurrence and metastatic spread remains a concern [14]. Favorable prognostic factors include an early stage of disease at presentation, the botryoid variant, and the absence of nodal or distant metastasis [16]. The pleomorphic or alveolar variants of RMS and deeply invasive disease are associated with a poor prognosis, increased risk of treatment failure, and disease recurrence [6, 16].

In conclusion, embryonal RMS of the uterine cervix is a rare, mesenchymal tumor that primarily affects adolescents and young adults. Clinicians should demonstrate a high index of suspicion for RMS when a young female presents with an enlarging cervical mass. A multimodal approach inclusive of surgical intervention, systemic chemotherapy, and radiotherapy remains the standard of treatment. In recent years, the focus has shifted from radical surgery to local cytoreductive procedures to preserve fertility, in applicable cases. Embryonal RMS is associated with a favorable prognosis, particularly when patients present with early-stage disease, and a multimodal approach to treatment is employed.

## Data Availability

Data including reports, patient details, and consent is stored on a secure drive accessible to the corresponding author.
